# Current approaches to gene regulatory network modelling

**DOI:** 10.1186/1471-2105-8-S6-S9

**Published:** 2007-09-27

**Authors:** Thomas Schlitt, Alvis Brazma

**Affiliations:** 1 Department of Medical and Molecular Genetics, King's College London School of Medicine, 8^th ^floor Guy's Tower, London SE1 9RT, UK; 2 European Bioinformatics Institute, EMBL-EBI, Wellcome Trust Genome Campus, Cambridge CB10 1SD, UK

## Abstract

Many different approaches have been developed to model and simulate gene regulatory networks. We proposed the following categories for gene regulatory network models: network parts lists, network topology models, network control logic models, and dynamic models. Here we will describe some examples for each of these categories. We will study the topology of gene regulatory networks in yeast in more detail, comparing a direct network derived from transcription factor binding data and an indirect network derived from genome-wide expression data in mutants. Regarding the network dynamics we briefly describe discrete and continuous approaches to network modelling, then describe a hybrid model called Finite State Linear Model and demonstrate that some simple network dynamics can be simulated in this model.

## Introduction

Most cellular processes involve many different molecules. The metabolism of a cell consists of many interlinked reactions. Products of one reaction will be educts of the next, thus forming the metabolic network. Similarly, signalling molecules are interlinked and cross-talk between the different signalling cascades forms the signalling network. And the same is true for regulatory relationships between genes and their products. All these networks are closely related, e.g. the regulatory network is influenced by extracellular signals. But there are characteristic features in the signalling network, which do not exist in the regulatory network; therefore dealing with these networks separately makes sense. Our main interest is in transcription regulation networks and we will refer to them as "gene networks", but many principles are valid for a wide range of networks. High-throughput technologies allow studying aspects of gene regulatory networks on a genome-wide scale and we will discuss recent advances as well as limitations and future challenges for gene network modelling. This survey is largely based on and is an extension of two previous publications [1,2].

**Figure 1:**
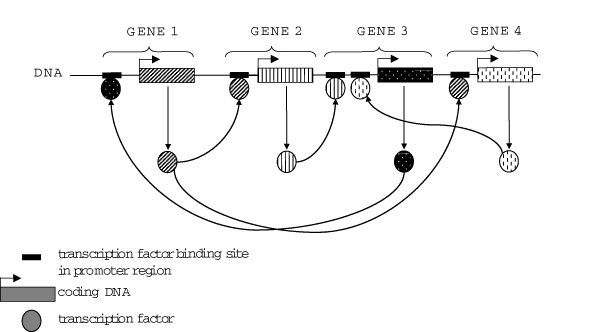
**Representation of a simple, fictional transcription factor network**. All genes shown encode transcription factors that control the activity of genes encoding transcription factors.

Gene networks are concerned with the control of transcription, i.e. how genes are up and down regulated in response to signals. In the 1960's genetic and biochemical experiments demonstrated the presence of regulatory sequences in the proximity of genes and the existence of proteins that are able to bind to those elements and to control the activity of genes by either activation or repression of transcription. These regulatory proteins are themselves encoded by genes (Figure 1). This allows the formation of complex regulatory networks, including positive and negative feedback loops. These principles of gene regulation apply to prokaryotes (e.g. bacteria) as well as to eukaryotes (e.g. higher organisms). The control of gene activity is much more complex than Figure 1 suggests. It involves many kinds of proteins thus allowing additional levels of control particularly in eukaryotes. Transcription factors, the proteins that recognize the regulatory elements in the DNA (the binding sites) need to interact with other proteins in order to activate gene expression. In addition to control of gene expression there are regulatory controls to determine the maturation, transport and degradation of the mRNA, as well as its translation. Just to illustrate the complexity of gene regulation: Gene Ontology (GO), a controlled vocabulary used to describe protein functions contains currently over 7500 different terms describing biological process 'transcription', including over 6500 terms under process 'regulation of transcription' [3].

Gene networks are often described verbally in combination with figures to illustrate sometimes-complicated interrelations between network elements. Due to the complexity of these networks, such models are not always easy to comprehend and they often leave a considerable amount to ambiguity to the reader's imagination. Since the 1960's methods from mathematics and physics have been used to describe and simulate small gene networks more stringently. Nowadays, molecular biological methods and high-throughput technologies make it possible to study a large number of genes and proteins in parallel enabling the study of larger gene networks. This allows tackling gene networks more efficiently and has led to a new discipline called Systems Biology, which seeks to combine methods from biology with methods from mathematics, physics and engineering to describe biological systems.

We proposed to categorize gene networks models in four classes according to increasing level of detail in the models [1]. Each class has its own advantages and limitations. The four classes are:

i. *parts lists *– a collection, description and systematisation of network elements in a particular organism or a particular biological system (e.g., transcription factors, promoters, and transcription factor binding sites);

ii. *topology models *– a description of the connections between the parts; this can be viewed as wiring diagrams where directed or undirected connections between genes represent different types of interactions;

iii. *control logic models *– a description of combinatorial (synergetic or interfering) effects of regulatory signals – e.g., which transcription factor combinations activate and which repress the transcription of the gene;

iv. *dynamic models *– the simulation of the real-time behaviour of the network and the prediction of its response to various environmental changes, external, or internal stimuli.

Obviously, for a fixed number of network elements each next level is more detailed and complex. But the size of the networks that we are able to model at each particular level is limited. Much larger networks can be described on topological level than on the dynamic level. In the following section we will discuss these classes in more detail.

## Organisational levels of gene network models

"All models are wrong, but some are useful". – George E. P. Box

### Parts list

Compiling the parts list is the first step in developing any model of some complexity and is not always a trivial exercise. Simple parts lists of genes, transcription factors, promoters, binding sites and other molecular entities are useful means for assessing the network complexity and for comparing different organisms. Such parts lists can be the result of a genome-sequencing and annotation project, where the complete DNA sequence of an organism is determined and all (or at least many) genes and proteins are identified. A parts list could also be represented as a database of regulatory elements or it could be ontology terms of transcription regulation processes assigned to a set of genes.

Comparing such lists from different organisms can provide an indication of the complexity of the transcriptional machinery or be used to predict the presence or absence of particular metabolic pathways [4-6]. The number of known and predicted transcriptional regulators in eukaryotic organisms varies from about 300 in yeast to about 1000 in humans (Table 1).

**Table 1:** Number of transcription regulators in different organisms

**Organism**	**number of genes**	**number of transcription regulators**
yeast	6682	312 (4.7%)
fly	13525	492 (3.6%)
human	22287	1034 (4.6%)

The number of genes and transcriptional regulators (genes annotated with GO term GO:0030528 "transcription regulator activity" for yeast (*Saccharomyces cerevisiae*) was taken from SGD  and for fly (*Drosophila melanogaster, DROM3*) and human (*Homo sapiens, NCBI 34 dbSNP120*) was taken from ENSEMBL

Many publications address the computational identification of transcription factor binding sites for instance by analysing promoter sequences of coexpressed genes [7]. One approach is to search for short sequences that are overrepresented in the promoters of a particular group of genes (e.g. clusters of coexpressed genes or sets of longer sequences known to bind a particular transcription factor) in comparison to the promoter sequences of all other genes. This approach obviously depends on the availability of the sequences for many genes and their upstream regions. Such an approach was applied in a cell cycle study in *Schizosaccharomyces pombe*, where Rustici *et al*. showed that the presence or absence of consensus binding sites in the promoter regions corresponds to the cyclic expression pattern of the genes [8]. Genes with a peak expression at similar cell cycle stage often share similar sets of consensus binding sites.

However, the exact promoter regions are usually unknown and even the transcription start sites are only known for a few genes. Baker's yeast (*Saccharomoyces cerevisiae*) has a relatively small genome with short intergenic regions, considering about 600–1000 bp upstream of the translation start site (ATG) appears to be a good approximation for the promoter regions. In higher organisms like vertebrates the intergenic regions and thus the putative promoter regions are much larger than in yeast, therefore the identification of regulatory elements in the DNA sequence by computational means has turned out to be rather elusive. Some studies have focused on the computational analysis of higher-level organisation of transcription factor binding sites in promoters, such as frequently occurring combinations of known binding sites [9,10], or restricted the search for regulatory elements to conserved sequence regions, identified by genome comparisons, a method often referred to as phylogenetic footprinting [11]. However, phylogenetic footprinting does not always work, because the localisation and the binding sites themselves are not always conserved [12,13].

Transcription factor localisations can be identified experimentally, too. For example, individual binding sites can be detected using the "DNAse I footprinting assay"; proteins bound to the DNA protect it from degradation by DNAse I, therefore these regions can be analysed further [14]. Another common experimental method is the "electrophoretic mobility shift assay" (EMSA) sometimes called "band shift assay" or "gel retardation assay" – DNA fragments that are bound by protein move slower in an electrophoretic gel than unbound fragments [15,16]. These methods allow fine mapping of individual binding sites, but are very labour intensive. High-throughput methods such as the Chip-on-chip method (see additional file 2, which describes this methods in more detail) allow the genome-wide detection of binding sites for a transcription factor, but the spatial resolution and signal quality is limited. Furthermore, assigning transcription factors to their target genes based on the genomic localisation is difficult due to the size of intragenic and intronic regions and long range effects of some transcription factors.

Nevertheless parts lists provide the first impression of gene networks in different organisms and they are necessary to have before we continue by having a look at the network topology.

### Topology models

Once we know the transcription factors and their binding sites, we can describe the gene transcription regulatory networks by graphs with nodes corresponding to genes and edges to regulatory interactions [17]. (Note the difference between these *discrete *graphs and plots of mathematical functions also often referred to as graphs.) A short introduction to discrete graphs is given in additional file 1, for more details please consult for example Cormen *et al*. [18]. One important concept that we will use below is a representation of a graph by a so-called adjacency matrix, where the element *a*_*ij *_in a row *i *and column *j *equals 1 (i.e., *a*_*ij *_= 1), if node *i *is connected to node *j*, otherwise *a*_*ij *_= 0. Graph representations have been used for various biological data sets ranging from protein-protein interactions networks to coexpression networks, they have been long used in mathematics, physics and computer science, and many aspects of graphs and their applications have been studied (e.g., [19-21]).

**Figure 2:**
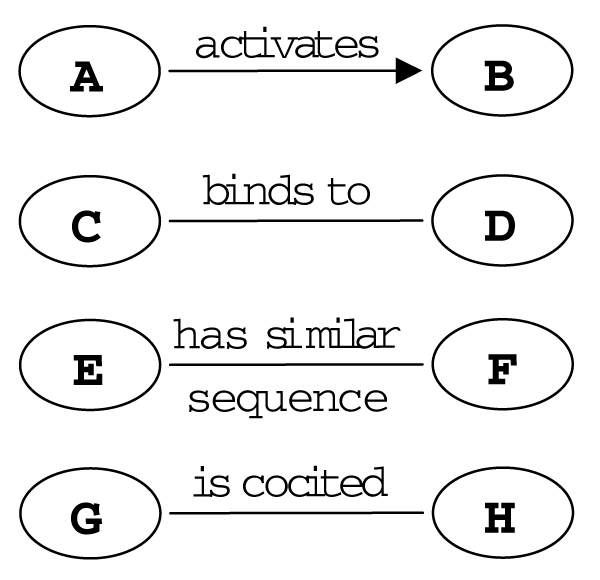
**Edges and arcs of a graph can represent different kinds of relationships**. Some examples are shown.

In a directed graph (i.e., a graph where connections between nodes have a definite direction) we call genes (nodes) with outgoing edges (arcs) *source genes*. For a given source gene, we call the set of all genes with incoming arcs from that source gene its *target genes*. Regulatory relationships can be of various natures. For a specific model we need to define the precise meaning that we assign to the edges (Figure 2). For instance, an arc from a gene *A *to *B *may mean that source gene *A *is a transcription factor, which is known to bind to the promoter of target gene *B*. A rather different network will be obtained if an arc from *A *to *B *denotes the observation that the disruption (e.g., mutation) of source gene *A *changes the expression of target gene *B*. We will present examples for these types of networks in the next section. A widely studied type of molecular network of a different type is the protein-protein interaction network, where the nodes represent proteins and two proteins are connected by an undirected edge, if they bind to each other (Figure 2) [22]. A different network will be established by connecting genes based on their sequence similarity. Networks can also be built based on the co-occurrence of gene names in journal abstracts. If two gene names frequently occur in the same abstracts it is likely that they share some kind of functional relationship [23].

To illustrate knowledge that we can obtain from studying network topology, we will compare and combine information from two high throughput data sets for the yeast *Saccharomyces cerevisiae*. The first is obtained from chromatin immunoprecipitation experiments for transcription factors (*ChIP network*), while the second is obtained from microarray experiments on single gene deletion mutants (*mutant network*), for more detail on the experimental methods please refer to additional file 2. Microarray experiment measurements can be presented in a data matrix, where rows represent genes and columns particular experiments (hybridisations). For instance, in the ChIP experiment each column will correspond to a particular transcription factor (studied in the particular experiment), while in the mutant experiment each column will correspond to a particular mutant. In this way, not only rows, but also columns will correspond to genes. The measurement values are typically real numbers, such as intensity levels, expression levels, or p-values, depending on the data processing steps applied. By applying an additional data processing step, often called thresholding, we can transform these continuous values into discrete values (e.g., if we chose a threshold *t*, then we can replace any *x*_*ij *_by 0, if *x*_*ij *_<*t*, and by 1, otherwise). In this way we will transform the original *measurement matrix *for these experiments into an adjacency matrix defining a graph: two genes in this graph are connected, if the measurement value is higher than the chosen threshold.

The ChIP network is based on experimental data published by Harbison *et al*. [24]. As described in additional file 2 they used genomic tiling arrays to identify the genomic regions bound by transcription factors. The authors assigned each genomic region to one or two target genes based on proximity in the genome. Relative intensities of spots are the basis for an error model that assigns a probability score (p-values) to binding interactions, which we use for discretisation.

The starting point for the mutant network is the gene expression data matrix published by Hughes *et al*. [25]. Each experimental condition, which in our case is a particular gene deletion mutant, corresponds to a column and each gene corresponds to a row (for more detail see additional file 2). We discretise the data matrix using a gene-specific standard deviation estimate γ obtained from the error model proposed by Hughes *et al*. [25].

The size of the networks depends on the discretisation thresholds chosen (Table 2). The criteria used to choose these thresholds are rather subjective, for the following comparisons we focused on these thresholds: ChIP network p_t _= 0.001 and mutant network γ = 2.5.

**Table 2:** Some properties of the mutant network and the ChIP network at different thresholds

	**ChIP network (p < 0.01)**	**ChIP network (p < 0.001)**	**mutant network (γ = 2.0)**	**mutant network (γ = 2.5)**	**mutant network (γ = 3.0)**
source genes	202	169	250	236	227
target genes	4939	2845	5396	4778	3920
genes	4980	2930	5654	4798	3959
edges	18842	6170	32017	17436	10356
edges where source gene and target gene have the same cellular role annotation in YPD	3694 (19.6%)	857 (13.9%)	4096 (12.8%)	2425 (13.9%)	1507 (14.6%)
edges per source gene	93.3	36.5	135.7	73.8	45.6

What do these two networks mean and how do they compare? In the ChIP network, an arc A→B means that the gene A codes for a transcription factor that binds to the promoter of gene B, while in the mutant network it means, that the mutation of A will change the expression level of B [23]. The ChIP network describes physical interactions, but it does not tell us anything about the effects of these interactions. The mutant network is similar to the one used in gene networks built by classical genetics means – we know that a mutation (perturbation) of the first gene has an effect on the second one, but it does not necessarily mean a direct physical interaction – there may be a long transcriptional or signalling cascade leading from the first gene to the second. In this way the first network is likely to contain direct interactions, while the second may include indirect interactions as well. However, it is possible that some of the 'direct' interactions of the Chip network are not biologically functional and thus may not be supported by mutant network. Most importantly it should be noted that the experimental conditions in the two experiments are not identical, which may result in considerable discrepancies between the two networks.

**Figure 3:**
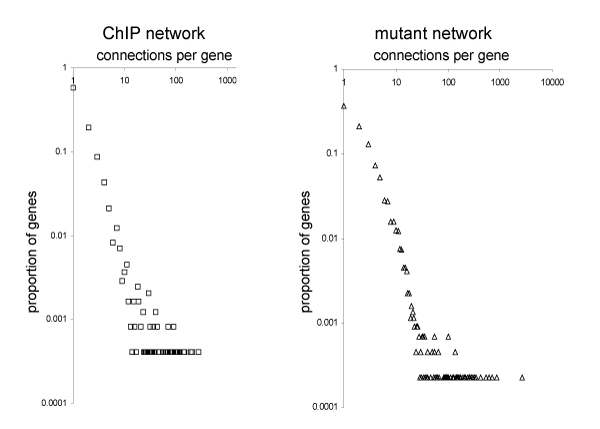
**Log-log plot of the node connectivity in different topological networks**. The genes with the highest degrees are *ABF1 *in the ChIP-network and *TUP1 *in the mutant network, adapted from [2]

First we can observe that both networks consist of one major component and almost all genes are part of it and are connected (this is true for a wide range of discretization thresholds). The degree distributions resemble roughly a power-law, i.e. most source genes have few target genes, while few source genes have many (Figure 3). Rung *et al*. discovered that the number of connections can indicate the functional class of the gene. Genes in the mutant network with many outgoing arcs (high *outdegree*) often encode proteins with regulatory functions, whereas genes with many incoming arcs (high *indegree*) are predominantly involved in metabolism [26]. Functionally related genes tend to be close in the networks, it is therefore possible to identify functionally related genes by comparing their neighbourhoods [23]. Manke *et al*. found directly interacting transcription factors and those, which are members of a protein complex, to occur together as putative DNA-binding modules more often than expected randomly [27].

**Figure 4:**
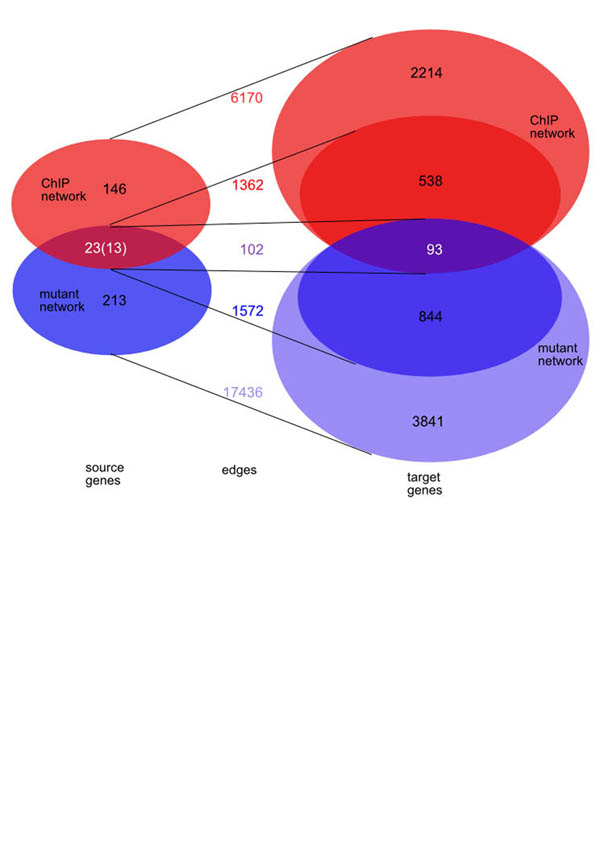
**Venn diagrams of the intersection between the mutant and the ChIP network**. The Venn diagram on the left hand side shows the intersection of the source genes between the mutant network and the ChIP network; the right hand side shows the intersections of the target genes between both networks. The connections between the two Venn diagrams indicate the corresponding number of edges. The networks share 23 source genes and 102 edges, but only 13 of the shared genes contribute to 102 shared edges, which connect to 93 distinct target genes. The 23 shared source genes are connected by 1362 edges to 631 target genes in the ChIP network and by 1572 edges to 937 target genes in the mutant network (see also Table 3).

**Figure 5:**
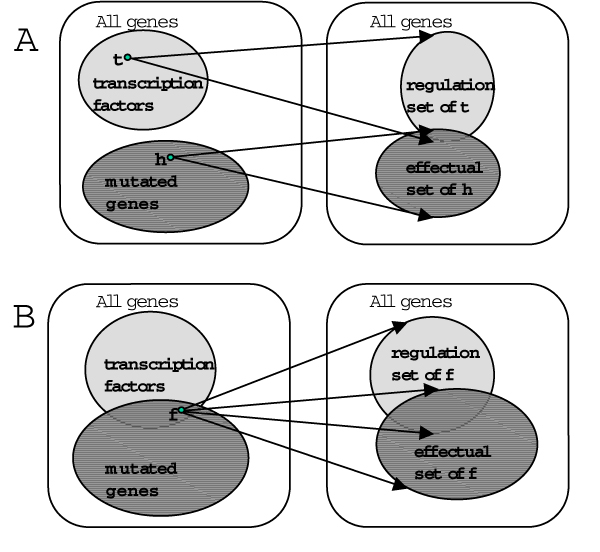
**Illustration of the target set comparison**. **A **In the ChIP network transcription factors are connected to their target genes (regulation set); in the mutant network the deleted genes are linked to all genes with differential expression in this particular mutant background (effectual set). **B **Some transcription factors are present in both networks (ChIP and mutant network); we can therefore compare the genomic localisation (regulation set) with the expression changes in the mutant cell (effectual set). Reproduced from [2]

**Figure 6:**
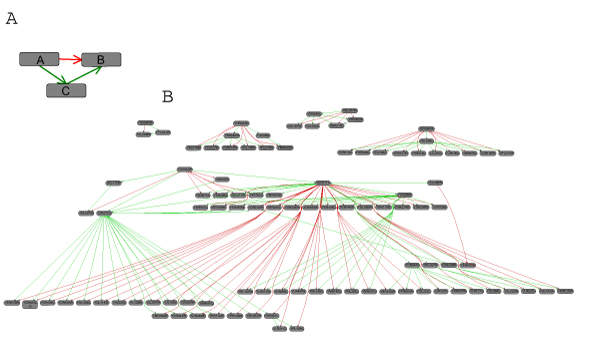
**Direct and indirect effects**. Red arcs are from the mutant network, green arcs from the ChIP network. A In the mutant where transcription factor A is deleted (disrupted) the expression of gene B is significantly different from its expression in the wild type. The transcription factor A does not bind to the putative promoter region of gene B (no green arc), but to the putative promoter region of transcription factor C, which in turn is found in the putative promoter region of gene B. This indirect path from A to B in the ChIP network might therefore explain the direct path in the mutant network. B All direct effects in the mutant network that could be explained by indirect paths via one additional transcription factor in the ChIP network.

When we compare and combine the mutant network and the ChIP network we immediately observe that their intersection is sparse. They share 102 edges connecting 13 from a total of 23 shared source genes to 93 distinct target genes (Table 3 and Figure 4). In the mutant network these 13 source genes are connected to 937 distinct target genes by 1157 edges (89 edges per source gene, 8.8% of the connections are in the intersection); whereas in the ChIP network they are connected to 631 distinct target genes via 924 edges (71 edges per source gene, 11.0% of the connections are found in the intersection). We used the hypergeometric distribution to compare target sets of all source genes [23] to identify source genes with target set intersections larger than expected by chance (Figure 5). We found that 9 of the 23 shared transcription factors Arg80p, Gcn4p, Hir2p, Mbp1p, Stb4p, Ste12p, Swi4p, Swi5p and Yap1p have significantly similar target sets in both networks (p < 0.05, 4000 genes in total) (Table 3). In these cases the transcription factor localisation might actually explain the changes in gene expression we see in the corresponding deletion mutants. One would assume that due to the nature of the experiments some effects in the mutant network are indirect effects that could be explained by a combination of direct connections in the ChIP network (Figure 6A). We find that indeed a number of connections in the mutant network can be explained by a combination of two edges in the ChIP network (Figure 6B).

**Table 3:** Degrees of the source genes that are shared between the mutant network and the ChIP network (data for YPD medium only)

		target genes			
source gene		in the mutant network	in the ChIP network	shared between ChIP and mutant network	intersection significant according to hypergeometric test
YAL051W	YAF1	1	61	0	no
YOR028C	CIN5	1	153	0	no
YHL009C	YAP3	2	18	0	no
YKL043W	PHD1	3	67	0	no
YLR014C	PPR1	7	25	0	no
YBR083W	TEC1	20	42	0	no
YMR275C	BUL1	27	3	0	no
YPL049C	DIG1	32	51	0	no
YLR113W	HOG1	145	12	0	no
YOL067C	RTG1	177	6	0	no
**YER040W**	**GLN3**	**52**	**16**	**1**	no
**YMR021C**	**MAC1**	**52**	**42**	**2**	no
**YGR040W**	**KSS1**	**253**	**18**	**2**	no
**YLR182W**	**SWI6**	**42**	**158**	**3**	no
**YOR038C**	**HIR2**	**25**	**16**	**2**	**yes**
**YMR042W**	**ARG80**	**5**	**16**	**4**	**yes**
**YDL056W**	**MBP1**	**6**	**134**	**4**	**yes**
**YMR019W**	**STB4**	**9**	**33**	**5**	**yes**
**YML007W**	**YAP1**	**37**	**72**	**8**	**yes**
**YDR146C**	**SWI5**	**35**	**120**	**9**	**yes**
**YHR084W**	**STE12**	**43**	**63**	**14**	**yes**
**YEL009C**	**GCN4**	**51**	**75**	**19**	**yes**
**YER111C**	**SWI4**	**547**	**161**	**29**	**yes**

sum of edges		1572 (**1157**)	1362 (**924)**	**102**	

13 source genes have common target genes in both networks (bold). Last column shows if the intersection between the target sets is larger than expected randomly (hypergeometric test, p < 0.05, 4000 genes in total)

Others have observed that when comparing different protein-protein interaction networks, their intersections are small, too. Only few interactions are reported by several experiments despite using just different methods to measure the same interactions [28]. Reassuringly, the shared interactions turned out to be more reliable than most of the data [28]. Here we work with experiments measuring different, if somehow related effects, but still the proportion of connections between functionally related genes is increased in the intersection of the two networks. In the intersection 40 of 102 (39.2%) connections connect genes that have the same cellular role in YPD, compared to less than 20% in the original networks (Table 2).

To summarise we can say that although the two networks are rather different, the part that is common to both is biologically more meaningful, and some of the indirect interactions of the mutant network can be explained by the direct interactions in the chip network.

Network topologies, particularly in yeast, have been widely studied and many interesting observations have been made. It has been proposed that the existence of highly connected genes (hubs) in a network might make networks more tolerant to random failure of network elements [29,30]. In protein-protein interaction networks it seems possible to classify hubs in combination with expression data: Han *et al*. [31] showed that hub proteins can be divided into two groups based on the level of coexpression between their neighbours in the network (the proteins directly connected to the hub proteins). Hubs with low coexpression seem to link functionally separate modules and removing these hubs leads to more rapid disintegration of the network [31]. However so far this has not been observed in transcription networks.

Luscombe *et al*. compiled data from ChIP-on-chip experiments for yeast to construct a network of 142 transcription factors, 3420 target genes and 7074 regulatory interactions [32]. To study the dynamics of this network they traced the paths from the target genes back to initial transcription factors, starting from target genes that are differentially expressed under particular conditions as demonstrated in previously published microarray experiments. Depending on the conditions, different sets of genes are expressed, leading to different sets of target genes as start points for the backtracking and different transcription factors along the path. This is consistent with results from ChIP-on-chip experiments and demonstrates that the topology of the gene regulatory network is not independent of the experimental conditions [24].

In a different line of investigations, Lee *et al*. [31], and Milo *et al*. [33], identified re-occurring structural elements (motifs) in the networks. They examined topological networks derived from ChIP-on-chip data for structures consisting of 3, 4 or more edges that occur in the original network more often than in randomised networks. Network motifs they identified to be significantly more frequent than in randomised networks included feed-forward and feedback loops. These motifs may partly be the result of gene duplications during genome evolution [34].

These are just some examples of possible analyses that can be performed on topology level. However, arguably the main reason to study the network topology is to prepare the ground for the next step of building more detailed models for gene networks. Before any logic or dynamic network model can be constructed we need to know which gene products interact and which are mutually independent. Even if we take the view that in the real-world network every gene is connected to every other gene to some degree, not all these connections are equally strong and a discretization step can be used to keep only the strong connections in the model. A complete network where everything is connected to everything may not be a practical approach to network modelling on a genome scale.

Arguably the most important question is if we can find modules, i.e. subnetworks that are relatively isolated from the rest of the network. If such modules are found, they can help us to use the reductionist approach later on by allowing modelling the parts of the network independently on a more detailed level. For instance, if we can build a dynamic model of an independent module, then we can perform simulations independently from the rest of the network. The existence of modules in biological systems has often been taken as an axiom [35]. However, a precise definition for what constitutes a module is elusive, and therefore this term has been used in various contexts [36]. In a graph representation it is natural to define a module as a 'relatively' isolated component, and indeed such components were found in protein-protein interaction networks. In contrast isolated components have hitherto not been found in the wiring diagrams of eukaryotic transcription regulation networks [26]. Several methods have been proposed to identify modules as groups of genes coexpressed under specific conditions [37,38], but there remain controversial opinions regarding the existence and nature of modules in gene networks [39,40]. Biologically meaningful pathways are sometimes used to define modules and to implement a reductionist approach. However, this easily breaks down in conditions when the pathways interact.

In general, data sets used for topological models have important limitations. While hundreds of organisms have been fully sequenced and many genes are identified relatively reliably, the data sets underlying most topological models are much less complete. Only a fraction of all protein-protein interactions in yeast have been tested; most large-scale experiments show high noise levels; and whereas the genome sequence is independent of particular growth conditions and (sometimes) is even conserved in fossils, data like protein-protein interactions and transcription factor localisations are condition dependent. In this context it is particularly important to note that some experimental methods are performed under conditions considerably different from the natural conditions in the cell. For example, the yeast-2-hybrid technology was used to determine protein-protein interactions between human proteins, yet the yeast cell provides a very different environment from a human cell [41,42]. This can be a considerable source for variation and systematic errors. Some experimental techniques are performed in test tubes, thus providing the most "unnatural" conditions. Unfortunately various limitations are unavoidable and we have to work with incomplete data for a limited set of conditions.

We can conclude that genome scale topological representations have helped us to make many interesting observations about the network properties, however the main question of finding well defined modules of such networks on topology level is still open.

### Control logics models

**Figure 7:**
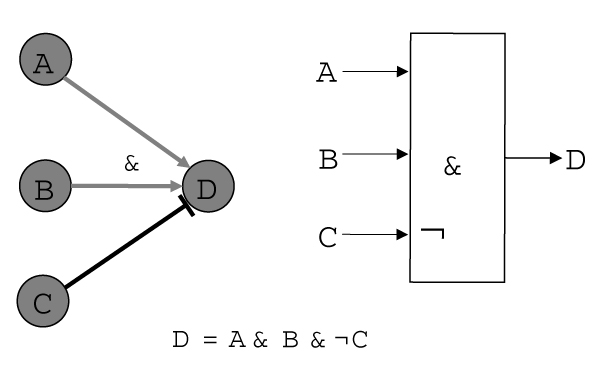
**Example for network logics**. Genes A, B and C control the activity of gene D; D is active if A and B are bound, but not C; right: shows the FSLM representation for such a promoter. Reproduced from [2].

Once we know the network topology, the next step is to study the rules of interaction between the different elements in the network. For instance, if a promoter consists of only one binding site for a transcription factor, we may want to know whether it is an activator or a repressor. If several transcription factors bind to a promoter, we need to know what each factor does, but also how these factors interact (Figure 7). Biological studies demonstrate that some promoters show combinatorial behaviour that can be approximated by Boolean functions (AND, OR, NOT and combinations of these), but in other cases the interaction is more complicated [43]. Linear functions, Boolean functions, decision trees, and Bayesian probability distributions have all been used to describe the network logic. We can distinguish between discrete functions and continuous functions. Discrete functions are based on the assumption that a gene can be in a finite number of states. In the simplest case we use only two different states to describe the activity of genes (e.g., *expressed *and *not expressed*). We can thus use Boolean functions to describe the interactions between transcription factors, e.g. "gene j is active, if transcription factor A AND B are bound to the promoter". It has to be stressed that such 'states' are only approximations of reality, that in the real world the interactions are not so well defined and are often fuzzy.

Continuous functions use continuous values (real numbers) to represent the gene activity. Weights *w*_*ij *_represent the interaction between genes *i *and *j*, which can be positive or negative. Thus the activity g_*i *_of gene *i *can be calculated as the weighted sum of the activities of all *n *genes:

*g*_*i *_= *w*_*i1 *_*g*_*1 *_+ ... + *w*_*in *_*g*_*n*_

This approach assumes that the influence of one gene on another gene is linear. Note that the network topology will determine which of the weights *w*_*ij *_are equal to 0 (i.e., if there is no arc from gene *i *to gene *j *in the network topology, then *w*_*ij *_= 0). Like Boolean functions, linear functions are only approximations. For instance, it is not possible by linear functions to model a situation where the same transcription factor can play a role of an activator or repressor for the same gene, depending on the presence or absence of other transcription factors.

Although few promoters have been studied in great detail, there are excellent examples, such as the description of the promoter action logics of sea urchin developmental gene *Endo16 *[44]. The *Endo16 *promoter consists of almost 30 regulatory elements stretched over a region of 2.3 kb. Based on experimental data collected using modified promoter constructs Davidson and co-workers constructed a model expressed as an algorithm combining Boolean and linear functions. This algorithm takes as an input the occupancy information from 12 binding sites and outputs a value, that 'can be thought of as the factor by which, at any point of time, the endogenous transcription activity (...) is multiplied as a result of the interactions mediated by the *cis*-regulatory control system' [44]. Predictions of promoter manipulations based on this model have largely been confirmed in subsequent experiments. Extending their earlier work the group of Davidson compiled a regulatory network containing over 40 genes by the construction of a model that integrates extensive experimental evidence on early development of sea urchin embryos [45].

Recently Klamt *et al*. published an example for control logic networks [46], based on hypergraphs, which are an extension to the graphs described above. Several hyper-edges pointing to the same node represent OR relationships, but edges are allowed to combine to represent AND relationships. Weights on the edges distinguish positive and negative relationships. The authors provide a set of methods to analyse these networks, just to list a few examples: computation of all positive and negative signalling paths, computation of all positive and negative feedback loops and computation of minimal cut sets. These minimal cut sets report the smallest number of interventions necessary to force the network into a particular behaviour, for example, a minimal number of deletions necessary to block the activation of a particular downstream protein in a signalling cascade. These methods are implemented in the software tool CellNetAnalyzer and the example presented, a model of a signalling network for T-cell activation shows that these analyses are non-trivial for signalling networks of a typical size.

**Figure 8:**
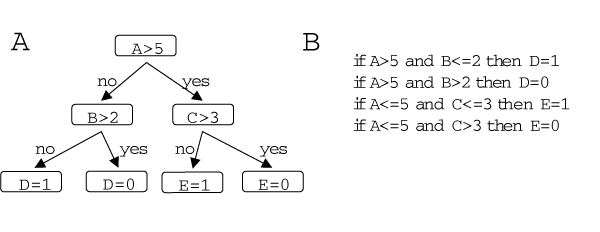
**Decision trees**. A decision tree is a special type of tree where the root and each interior node correspond to a variable; an arc to a child represents a possible value of that variable. A leaf represents the predicted value of target variable given the values of the variables represented by the path from the root. Following a route from the root node to a leaf node at each interior node we have to decide, which path to follow. Effectively each possible path encodes a decision rule. **A **Example for a decision tree. By following from the root node (top) to a leaf node (bottom) one has to make a decision at every interior node. **B **Corresponding set of decision rules.

Soinov *et al*. used a supervised learning approach to build decision-tree-related classifiers. A decision tree is a predictive model (Figure 8). Soinov *et al*. built decision trees which allow us to predict the gene expression activity of a particular gene (leaf node) based on the expression data of other genes (interior nodes) [47]. Although the gene predictions are binary in this approach (the gene is predicted be "active" or "inactive"), this system utilizes continuous expression values, such as microarray data.

Bayesian networks provide a probabilistic framework for modelling gene regulatory networks [48-50]. Their graphical representation is a directed acyclic graph, where each node represents a variable and the edges represent dependencies. For a more detailed description of the application of Bayesian networks in gene expression analysis see the reviews by Pe'er [51] and Friedman [52]. Segal *et al*. applied a learning procedure based on probabilistic graphical models to networks consisting of groups of coregulated genes [53].

There are situations where neither Boolean rules nor linear functions are powerful enough to express the control logics: transcription factors might bind competitively, if one factor is bound, the other one is excluded, as is the case for example in the phage λ switch between lysis and lysogeny [54]. In some cases, transcription factors have to form homodimers or heterodimers to be fully functional. The transcription factors might have to bind sequentially or might act synergistically. In these situations it might be necessary to use more complex functions (here this would be solved by Boolean circuits with memory or delay). It remains an open question what is the minimum repertoire of functions to describe regulatory logics.

### Dynamic models

The knowledge of the parts list of a network, its topology and the control logics are necessary requirements in order to expand the model to capture dynamic changes during time. Compared to the approaches above, the dynamic models can be described as 'classical' approaches to gene network modelling, as many of them have been developed and studied long before the current *genome era*. Typically, they are relatively small, involving only a few genes. They aim at describing and often simulating the dynamic changes in the state of the system and predicting the network's response to various environmental changes and stimuli.

Various dynamic models have been proposed. Greller and Somogyi subclassified them [55] as follows: "Dichotomies for framing our thinking on how to best represent a particular biological network problem include the following distinguishing attributes: quantitative versus qualitative measurements; logical versus ordinal variables (e.g. Boolean versus abundances); deterministic versus probabilistic state transitions (e.g. differential equations versus hidden Markov); deterministic versus statistical overall system description (e.g. vector field versus Bayesian belief network probability distributions); continuous versus discrete state (e.g. continuous intensities or concentrations versus low, medium and high); nonlinear versus linear elementary interactions and state update rules (e.g. multiplicatives, sigmoids or non-monitonics versus linear ramps); high-dimensional versus low-dimensional (e.g. >> 100s of variables versus << 100 variables); stochasticity present and profound versus absent or present as nuisance noise (e.g. probabilistic state transitions versus small amplitude errors); measurement error substantially corrupting and obfuscating versus negligible distortion." In the following sections we describe several approaches following the discrete to continuous model axis. The discrete model approaches we consider include Boolean network based models [56-58] and Petri nets [59-62], the dynamic systems are based on difference or differential equations [63-65]. We will then discuss hybrid models, which combine discrete and continuous elements [66-68].

#### Boolean models

**Figure 9:**
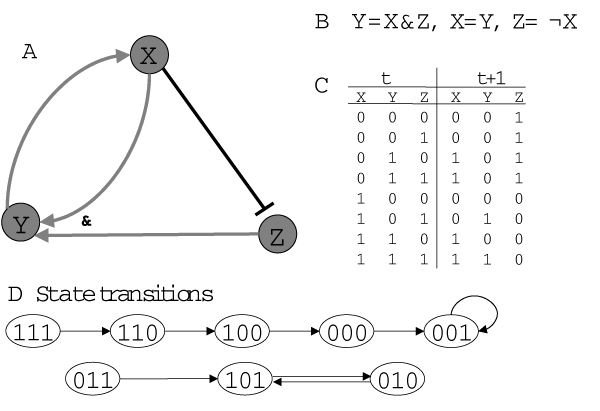
**Example for a small Boolean network **consisting of 3 genes X, Y, Z. There are different ways for representing the network: A as a graph, B Boolean rules for state transitions, C a complete table of all possible states before and after transition, or D as a graph representing the state transitions. Reproduced from [2].

The simplest dynamic models – *synchronous Boolean network models *– were used as a model for gene regulatory networks already in the 1960's by Stuart Kauffman as [69]. Boolean networks are based on the assumption that binary on/off switches functioning in discrete time steps can describe important aspects of gene regulation. In synchronous Boolean network models all genes switch states simultaneously (Figure 9). We can introduce the concept of the *state of the network *defined as an n-tuple of 0s and 1s describing which genes in the network are or are not expressed at the particular moment (Figure 9). As time progresses, the network navigates through the 'state space', switching from one state to another, as shown in Figure 9D. For a network of n genes, in total there are 2^n ^possible different states, for instance, for a three gene network the possible states are (0,0,0), (0,0,1), ..., (1,1,1). We can follow the succession of states with time and study which states are reached. Some states might never be reached. It is possible to look for attractors: these are states or series of states that once reached will not be left anymore. The small example network in Figure 9 has two attractors: one attractor is a single state (0,0,1), and the second attractor consists of two alternating states (1,0,1) and (0,1,0).

Kauffman introduced the notion of *canalizing function *– a Boolean function that has at least one input variable (canalizing variable) and one value (0 or 1) for that input (canalizing value), which determines the value of the output of the function regardless of other variables (i.e., if the canalizing variable has the canalizing value, then the output of the function do not depend on other variables, but if the canalizing variable does not have the canalizing value, then the output of the function is determined by the values of other variables) [70]. He hypothesized that genes are predominantly controlled by such functions (whether this is indeed true is still unknown). Kauffman used randomly generated networks to study their general features [69]. He found that under certain assumptions about the network topology (a limited number of incoming connections at each node) and logics (promoters are predominantly controlled by canalizing functions) there are only a small number of states in which the network will stay for most of the time. These states are called *attractors*; any other state, if possible at all, will lead to an attractor state in a relatively small number of steps. Moreover, the system either reaches a steady state or fluctuates between the attractor states in a regular fashion. Kauffman hypothesized that attractors correspond to different cell types of an organism. The number of cell types predicted by this model corresponds well with our current knowledge [70].

This approach has been generalized in a number of ways. Randomly generated networks are used to study the dynamics of complex systems [71]. Stochastic extensions to deterministic Boolean networks were proposed – so-called noisy networks by Akutsu *et al*. [72] and Probabilistic Boolean Networks by Shmulevich *et al*. [73].

Thomas and Thieffry describe a generalized model for the qualitative description of gene regulatory networks [74]. They introduce a notion of gene state and image, the last effectively representing the substance produced by the respective gene. There is a time delay between the change of the gene state and the change of the image state. By introducing several levels of gene activity and thresholds for switching the gene states they go beyond binary models, but they do not make continuous changes possible.

#### Petri nets

**Figure 10:**
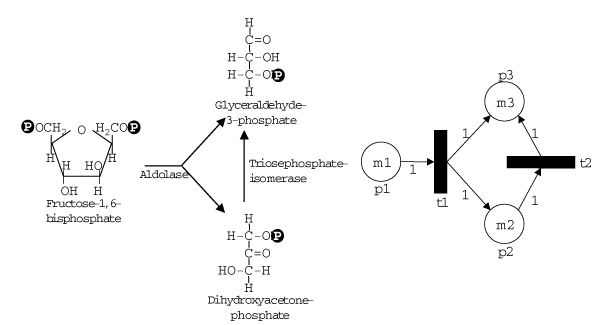
**A metabolic reaction (left) and its representation as a Petri net (right)**. Aldolase splits one molecule of Fructose-1,6-bisphosphate into one molecule Dihydroxyacetonephosphate and one molecule Glyceraldehyde-3-phosphate. The Triosephosphateisomerase then transforms one molecule Dihydroxyacetonephosphate into one molecule Glyceraldehyde-3-phosphate (the reversibility of the reaction has been omitted here for the sake of clarity). In the Petri net representation place nodes (circles) are denoted by p, transition nodes (boxes) by t and tokens numbers by m. The place node p1 represents Fructose-1,6-bisphosphate and m1 the number of tokens or number of Fructose-1,6-bisphosphate molecules present. The transition node t1 represents the enzyme Aldolase. The weights on the edges reflect the stoichiometry of the reactions. p2 Dihydroxyacetonephosphate, m2 number of Dihydroxyacetonephosphate molecules, t2 Triosephosphateisomerase, p3 Glyceraldehyde-3-phosphate, m3 number of Glyceraldehyde-3-phosphate molecules.

Petri nets are an extension to graph models and have been used successfully in many areas for example to simulate metabolic networks. For a brief introduction into Petri nets see Pinney *et al*. [59] or the more detailed reviews by Moore *et al*. and Hardy and Robillard [60,61]. Petri nets allow simple quantitative representation of dynamic processes like mass flow in a network. Petri nets were developed in the 1960's by Carl Adam Petri and have since been extended. In general they are directed graphs consisting of arcs and two different kinds of nodes, the place nodes and the transition nodes (Figure 10). The arcs only connect place nodes to transition nodes and vice versa. The dynamic aspect is introduced by so-called *tokens*. Each place node can contain tokens. Each arc has a 'weight' that determines how many tokens are needed for a transition along this arc. Intuitively, you can imagine that the tokens travel along the arcs if there are a sufficient number of them at the source node (as determined by the weighted arcs) and the transition nodes determine the exchange ratio along the way. In the simplest case, a transition node fires (= a transition takes place) always if sufficient tokens are present in the input place nodes.

In metabolic networks the place nodes represent metabolites and the transition nodes represent reactions. Metabolite concentrations correspond to the number of tokens in the particular place nodes and the stoichiometry is described by the weights of the arcs. Subsequent analyses of Petri net models look for place nodes running out of tokens or accumulating tokens and for subnetworks that are inactive. Interesting are invariants, such as transition invariants (T-invariants), where the transitions reproduce a given state. In metabolic networks T-invariants represent reactions reproducing the given concentrations of metabolites, as for example in steady state situations. For examples of the application of Petri Nets in the analysis of metabolic networks see articles by Koch *et al*., Kuffner *et al*., Schuster *et al*. or Steggles *et al*. [62,75-77].

Petri nets are particularly suitable for modelling metabolic reactions, because the similarities are intuitive and there is no need for detailed information about the reactions rates. This is an advantage, because often these rates are not known and hard, or at least costly to obtain. The lack of information about reaction rates is one major shortcoming for the application of differential models, which we will discuss in the next section. However, sometimes the reaction rates will be crucial to the function of the whole metabolic pathway and therefore need to be included in the pathway model (there are extensions to Petri Nets which address this, see the section on hybrid models below).

#### Difference and differential equation models

Boolean networks and Petri nets can reveal important network properties, but are too crude to capture some important aspects of network dynamics. Difference and differential equations allow more detailed descriptions of network dynamics, by explicitly modelling the concentration changes of molecules over time [63,64,78-80].

The basic difference equation model is of the form

g_1_(t+Δt) - g_1_(t) = (w_11 _g_1_(t) + ... + w_1n _g_n_(t)) Δt

...

g_n_(t+Δt) - g_n_(t) = (w_n1 _g_1_(t) + ... + w_nn _g_n_(t)) Δt

where *g*_*i*_*(t + Δt) *is the expression level of gene *i *at time *t + Δt*, and *w*_*ij *_the weight indicating how much the level of gene *i *is influenced by gene *j (i,j = 1...n)*. Note that this model assumes a linear logic control model – the expression levels of genes at a time *t+Δt*, depends linearly on the expression levels of all genes at a time *t*. For each gene, one can add extra terms indicating the influence of additional substances [64].

Differential equation models are similar to difference equation models, but follow concentration changes continuously, modelling the time difference between two time steps in infinitely small time increases, i.e. *Δt *is approaching 0.

Dynamic networks models have been reviewed intensively [81-84]. One of the largest transcription network models using differential equations we are aware of is a model for segment polarity genes and pattern formation in the early development of *Drosophila *by von Dassow *et al*. [85]. Their system included 48 parameters, such as the half-lives of messenger RNAs and proteins, binding ranges and cooperativity coefficients. The initial model described all known interactions, but it also revealed that the addition of at least two new hypothetical interactions were needed to ensure that the behaviour of the model was consistent with the observations.

Difference and differential models depend on numerical parameters, which are often difficult to measure experimentally. An important question for these models is *stability *– does the behaviour of the system depend on the exact values of these parameters and initial substance concentrations, or is it similar for different variations. It seems unlikely that an unstable system represents a biologically realistic model, while on the other hand, if the system is stable, the exact values of some parameters may not be essential. For instance, the *Drosophila *developmental model [85] is stable – it tolerates tenfold or more variation in the values of most individual parameters.

Many software packages have been developed for dynamical simulation of biological networks, but the exchange of models and data between these software packages was often not easy. The systems biology markup language SBML was developed to address this problem. SBML is an XML-based format that allows describing models software-independently [86]. (XML eXtensible Markup Language allows to define special-purpose markup languages, capable of describing many different kinds of data.) An example for a markup language is HTML. Nowadays SBML model descriptions can be used on many software platforms, enabling data exchange and cross-validation of models. This has also enabled the establishment of model databases like BioModels, a curated database of published quantitative kinetic models [86]. This is a central database where biological models published in scientific journals can be deposited.

#### Hybrid models

In the real world systems both continuous aspects and discrete aspects are present. In general, concentrations are expressed as continuous values, whereas the binding of a transcription factor to DNA is expressed as a discrete event (bound or unbound). However, the boundaries between the discrete and continuous aspects depend on the level of detail that our model is designed for. For instance, on single cell level the concentrations may have to be expressed by molecule counts and become discrete, whereas if we use thermodynamic equilibrium to model the protein-DNA binding, the variable describing the binding state becomes continuous.

Hybrid models have been developed in an attempt to describe both, discrete and continuous aspects in one model, and such models have therefore been proposed, for instance in [67,68].

There are extensions of the Petri net model, that allow us to include knowledge about the dynamics of reactions: for example to include stochastic time delays for the transitions, first applied to molecular biology by Goss and Peccoud [87]. In these networks the firing of transition nodes depends not only on the number of tokens in the input place nodes, but also on a stochastic component. In their study of circadian rhythms Matsuno *et al*. used another type of extension to Petri nets to simulate gene regulatory networks [88]: in addition to standard Petri nets Hybrid Functional Petri Nets (HFPN) contain continuous place nodes and continuous transitions. Continuous place nodes can hold a real numbers and continuous transition nodes are firing at a constant rate. In metabolic networks this rate corresponds to reaction rates. However, this means we loose one major advantage of Petri nets over difference and differential models: we need information on reaction rates. If we have information only for some reactions, HFPNs provide a compromise by allowing the implementation of a mix of continuous and discrete place nodes and transitions.

Another example for hybrid models is the phage λ model by McAdams and Shapiro [89], where elements similar to ones used to describe electronic circuits have been exploited.

#### Finite State Linear Model (FSLM)

As an example we will describe the *finite state linear model *(FSLM), more detailed descriptions of FSLM can be found in [2,90,91]. It combines the advantages of Boolean networks such as simplicity and low computational cost, with the advantages of continuous models, such as continuous representation of concentrations and time. The activity of genes is described by discrete states (e.g., gene is 'on' or 'off'), but the gene product concentrations are expressed as real numbers. Time is continuous in FSLM and the state of the network determines directly the concentration change rates, while the state is in turn affected by the concentrations themselves.

**Figure 11:**
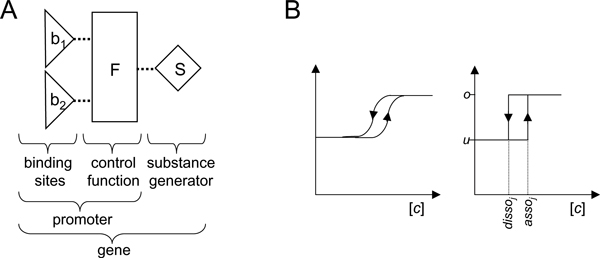
**The building blocks of the finite state linear model**. **A **Binding sites are represented by triangles, control functions by boxes and substance generators by diamonds. Dotted lines represent cases where the discrete output of one element is the input for another element. **B **Switching behaviour of the binding sites. The curve (left) is typical for processes with hysteresis characteristics of a system that does not instantly follow the forces applied to it, but reacts slowly, or does not return completely to their original state: that is, systems whose states depend on their immediate history. The threshold for switching the states of the binding sites in FSLM is state dependent and results in a similar curve (right). [*c*] concentration of substance binding to binding site *j*; *asso*_*j*_, *disso*_*j *_association and dissociation constants for binding site *j*; *u *binding site not occupied, *o *binding site occupied. Reproduced from [2].

**Figure 12:**
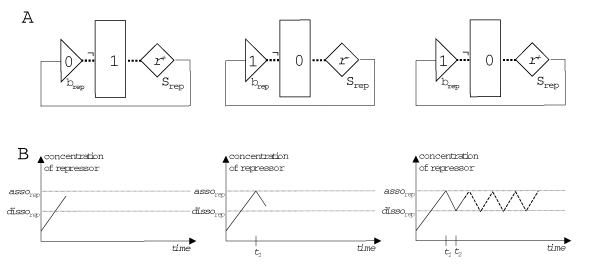
**Example for the dynamics of a simple FSLM network**. **A **In this negative feedback loop the substance generator produces a substance, which acts as a repressor of its own control function. **B **Environment change graph recording the changes in repressor concentration during time. From the initial concentration the repressor accumulates with rate *r*^+ ^until the association constant of the binding site *b*_*rep *_is reached at time *t*_1_. Then the substance generator is switched off and the repressor degrades with rate *r*^- ^until the dissociation constant is reached at time *t*_2. _The substance generator then produces the repressor until the association constant is reached again (means Boolean „not“). Reproduced from [2].

In FSLM there is only one class of molecules, represented by *substances*. There are three types of network elements: *binding sites, control functions *and *substance generators *(Figure 11A). The *binding sites *in the FLSM are comparable to DNA binding sites for transcription factors in the promoter regions of genes. A combination of binding site(s), control function(s) and a substance generator in the FSLM corresponds to a biological gene (Figure 11A). A gene network consists of one or more such genes, which influence each other via the substances they produce (Figure 12).

The binary FSLM allows only two possible states for the binding sites (*bound *or *unbound*) and substance generators (*on *or *off*). A generalisation of the FSLM model and a more mathematically thorough definition can be found in [91]. For each *substance *there is a corresponding *substance generator*. The substances can bind to *binding sites*, but each binding site can be bound by one substance only. The binding of a substance to a binding site *b*_*j *_depends on the *association constant a*_*j *_and the *dissociation constant d*_*j *_of the binding site *(0 < d*_*j *_<*a*_*j*_*)*. The binding site is *bound *if the concentration of the binding substance exceeds the association constant. If the substance concentration falls below the dissociation constant then the binding site is released and switches to the *unbound state*. The biochemical equivalents of the association and dissociation constants in FSLM are affinity constants. The difference between the association constant *a*_*j *_and the corresponding dissociation constant *d*_*j *_leads to a hysteresis characteristics (Figure 11B) for the switching between the states of a binding site (see for example [65]). The concentration threshold for the switch between the states of the binding site depends on the state of the binding site itself. Using discrete states to represent the binding sites means we approximate the binding equilibrium with a simpler step function.

The states of a set of binding sites comprise the binary input vector to a Boolean *control function F*. Depending on the input state vector the control function computes an output state (*on *or *off)*. A *substance generator S *changes the concentration of a substance in time in a linear fashion. The concentration can either increase with rate *r*^+ ^or decrease with rate *r*^- ^(*r*^-^* < 0 < r*^+^), corresponding to substance production and degradation, respectively. The output state of a control function determines the activity of a substance generator, i.e. whether the concentration of a particular substance is increasing or decreasing. Note that the linear increase and decrease rates that are assumed in the FSLM are only approximations to the reality.

**Figure 13:**
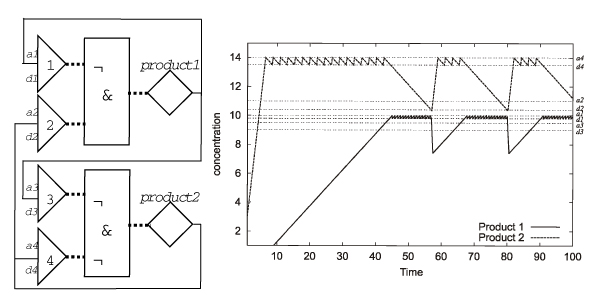
**A FSLM network consisting of two genes and four binding sites**. **Left: **The control functions of both genes have two inputs each. One input is from a binding site for its own substance, thus each gene is autoregulated by a negative feedback loop. *Gene 1 *has an additional negative feedback on *gene 2*, whilst *gene 2 *has an additional positive feedback on *gene 1*. **Right: **Result of the simulation of this network in FSLM. *a1 *association constantof *binding site 1*, *d1 *is the corresponding dissociation constant; *a2, d2, a3, d3, a4, d4 *correspondingly; ¬ Boolean *„not“*, *&*Boolean *„and“*. Reproduced from [2].

Let us illustrate the dynamics of the FSLM by modelling a negative feedback loop (Figure 12). To begin with the substance concentration of the repressor is low, the binding site is unbound, the substance generator is active and therefore the substance is produced with rate *r*^+^. Its concentration increases until it reaches the association constant of the binding site. The binding site switches to the bound state, which in turn leads to the inactivation of the substance generator, and the substance concentration decreases with rate *r*^- ^until it reaches the dissociation constant of the binding site. Consequently, the binding site switches to the unbound state, the substance is generated again, its concentration increases and the process repeats itself. Figure 13 shows the behaviour of a gene network consisting of two genes, demonstrating that a very simple network of just two genes can exhibit a non-trivial behaviour.

**Figure 14:**
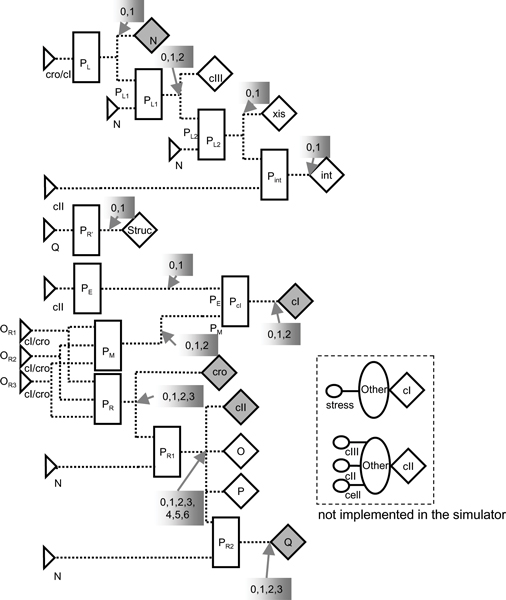
**Description of phage λ using the elements of FSLM**. In the FSLM model for phage λ the substance generators highlighted in grey produce substances, which bind to binding sites on the left (the connections have been omitted to improve the readability of the figure). The promoters P_*L*1_, P_*L*2_, P_*R*1_, and P_*R*2 _are used to model the behaviour of the λ terminator sites *t*_*L*1_, *t*_*L*2_, *t*_*R*1_, and *t*_*R*2_. The substance generators connected to them are only active, if N is bound to the respective binding sites. The substance "Struc" represents the structural proteins of the phage particles. The shaded grey boxes indicate the number of different states that the corresponding control functions can have. A simulation of phage λ using this model leads to lysogenic behaviour or lytic behaviour. In the *lysogenic mode *the initially active genes are inactivated, and the substance concentrations decrease rapidly, only CI is produced. The fluctuations of the CI concentration are due to the negative feedback loop involving the binding site O_*R*3_. In the *lytic mode*, CI and CII are not produced, but the other substance generators are active. The concentrations of Int, N, and Q increase infinitely because of the lack of a negative feedback control. The inset describes the effect of the stress response of the host cell using elements not yet implemented in the FSLM simulator. For a more detailed description of the model see [2, 91]. Reproduced from [2].

FSLM can be used to build complex models for instance to simulate the life cycle of phage λ (Figure 14). Phage λ is a virus that infects *Escherichia coli *cells [92]; it either integrates into the host genome and stays dormant (lysogenic) or causes production of new phage particles and lysis of the host cell, to allow spreading the infection. The decision for one or the other alternative (lysis vs. lysogeny) is made by the so-called lambda switch, which is based on competitive binding of two transcription factors to overlapping regions in the genome of phage λ. If the repressor is bound, the phage stays dormant, if the repressor is degraded and the activator can bind, new virus particles are being made. The FSLM model of phage λ allows two different kinds of behaviours, which correspond to lytic or lysogenic behaviour.

In our example models the biological systems are relatively simple, but for larger networks we often lack detailed information about the biology.

#### Stochastic networks

All networks mentioned so far are deterministic – they assume that the next state of the system is determined by the current state and the external inputs. However, in real world systems stochastic effects may play an important role. For instance, for some genes in yeast the number of mRNA molecules is close to one copy per cell [93]. This means that it is likely that there is a considerable intrinsic noise element present – some cells apparently have more mRNA molecules of the given species present than others. Thus modelling a cell by using continuous concentrations effectively means modelling an ensemble of cells by mean values of stochastic variables. It is not obvious to what extent this is possible. It has been demonstrated that the stochastic effects are important for the phage λ switch decision between lysis and lysogeny [94]. Lately experimental studies have tried to measure the level of intrinsic noise in eukaryotic cells (e.g., [95,96]). Simulating a stochastic model is computationally more expensive, because the simulations have to be run several times to provide a good impression of the system behaviour. But stochastic models are not always necessary; it depends on the system that is to be modelled. If the number of molecules involved is small and if important processes depend on random effects, stochastic models might be the best choice.

## Reverse engineering and synthetic networks

"With four parameters I can fit an elephant, and with five I can make him wiggle his trunk" – John von Neumann

### Reverse engineering of gene networks

*Reverse engineering *refers to an approach where one starts from data and tries to design a model that fits the data (semi-) automatically within the given model class, without additional prior hypothesis about the biological system. The model derived from the data is judged by the results of simulations compared to new experimental data. For example, one could use a gene expression data set to construct a particular gene network model that is consistent with the data. Inconsistencies between simulated data generated using this model and new data, that has not been used to construct the model, indicate shortcomings of the model. These inconsistencies can be used to choose between alternative models, or to improve the model. However, reverse engineering is possible only (1) if we have chosen an appropriate model class (in the sense that the desired properties of the real world network can be described in it), and (2) if we have enough quantitative data describing the behaviour of the system. Of course, even if the answers to these two questions are positive, reverse engineering is still a difficult problem, and few efficient algorithms are known. The methods chosen for reverse engineering depend crucially on the kind of modelling technique used. Quantitative models are normally more demanding than qualitative models. Dynamic models contain many parameters, and detailed experimental data are required to work out the parameters.

Miyano *et al*. have proposed algorithms to infer Boolean networks [67,72] and Friedman *et al*. developed methods to extract probabilistic graphical models, such as Bayesian networks from experimental data [49,52]. Tegner *et al*. proposed an approach for the reverse engineering of dynamic gene networks based on integrating genetic perturbations [97]. They identified " [...] the network topology by analysing the steady-state changes in gene expression resulting from the systematic perturbation of a particular node in the network." [97]. However, they only apply their approach to simulated data and to a comparatively small biological system consisting of only 5 genes.

### Synthetic networks

A powerful approach to test our understanding of gene regulatory networks is to build new networks from scratch in an approach called *synthetic biology*. Predictions of small models have been successfully tested experimentally using specifically engineered control circuits, such as feed forward loops [98] and feedback loops [99-103]. In a sense this is reverse engineering of a real world network. For a more detailed description see the reviews by Kaern *et al*. and Ball [104,105].

## Summary and open questions

"If you torture the data long enough, Nature will confess." – Ronald Coase

At the basis of any modelling, including network modelling, there is a realisation and acceptance that a model describes only some properties of the 'real world' system, and ignores others. Thus it emphasizes particular aspects of reality, leaving out details that are not relevant for the purpose of the study.

How far are we from being able to build realistic cell models? The availability of large-scale data sets such as microarray gene expression and genomic localisation data triggered the search for suitable approaches to model complex biological systems. As the result of genome projects we are now able to compile parts lists on genome scale, though we do not know how many important categories in these parts lists are missing. Models describing the network topology are approaching the whole genome scale. High-throughput experiments, most notably microarrays, provide us with temporal information about transcriptional processes in time series experiments. These have been used to study control logics as well as some dynamics aspects of transcription regulation in processes such as the cell cycle [8,106,107], stress response [108,109], or galactose utilization [110]. Models have been built to explore the fundamentals for example of the cell cycle for yeast [65] and improvements in the understanding of genome wide dynamics of cell cycle have been made [111]. Nevertheless, high-throughput technologies have yet to have a direct impact on quantitative real time simulations of gene networks.

The function of about one third of all genes is still unknown for the yeast *Saccharomyces cerevisiae *despite it being one of the best-studied organisms. And even for many of the better-known genes and core processes that have been studied for decades, like the cell cycle, there is still not enough data available to exactly know all changes in concentration and activation patterns. Currently it seems not feasible to simulate even relatively simple cells like yeast. Mechanisms like RNA interference, regulated degradation of mRNAs and proteins, chemical modifications of key molecules and others might play a larger role than anticipated in current models, other processes might still be unknown. It is obvious that the separation into gene regulatory networks, metabolic networks and protein interaction networks is possible only up to a certain degree. To what extent can the transcription regulation networks be decoupled from other networks, such as signal transduction networks? We need to integrate many types of information if we want to build realistic dynamic models, however, for current modelling approaches we have to limit the complexity of the systems we are dealing with.

One possibility to reduce the complexity of biological systems depends on the modularity of the real world networks and their robustness (stability against changes of various network parameters and initial conditions). If the networks are modular and robust, it might be possible to build genome scale networks as sets of smaller modules. If we can find modules – units behaving independently of each other – it would be possible to build the complete model as a set of modules.

The belief that real world biological networks 'must be' robust and 'must be' modular is quite wide spread. However precise definitions of biological robustness and modularity and, moreover, the proofs of their presence remain elusive. The principles of modularity and robustness used in engineering are sometimes given as a reason that the same must be true in biological systems, but there are many examples when the 'designs' in nature, which are obtained by natural selection are different from the designs one would use in engineering. However, there are other arguments why biological networks could be modular, such as reuse of the components after genome duplications, but they are no proofs. There are indications that, on the dynamic level, network modules exist. For instance, cell growth can be decoupled from cell cycle in yeast (e.g., [112]), indicating that to some extent independent modules control these two processes. Similarly, the *Drosophila *developmental network indicates that the exact values of the model parameters may not be crucial in large-scale systems behaviour [85]. But to what extent can specific processes be decoupled from each other?

Another possibility to reduce complexity in network models depends on the importance of the exact values of parameters and substance concentrations. How much do the exact quantitative values, such as substance concentrations, matter in determining the more general patterns of system behaviour, such as cell differentiation? If we are not interested in predicting the exact concentrations of different substances, but only in the patterns of the systems behaviour such as steady states, we can often use simplified Boolean-type networks instead of differential equations [113] and hybrid models might offer "good enough" solutions.

The question "Is real time simulation on genome scale possible at all?" is still open. Obtaining high quality systematic quantitative data characterizing systems parameters such as mRNA, protein and metabolite concentrations, interactions and spatial and temporal localization of different molecules will be important. Nevertheless, the data will not provide new insights automatically. We believe that hypotheses expressed as rigorously defined models, the properties of which can be studied independently and tested on experimental data, will play an important role in understanding the living systems on genome-wide level. In any case, finding the right language for describing the models is a prerequisite for success.

## Supplementary Material

Additional File 1 A short primer on graph theory Click here for file

Additional File 2 A very short primer on biology techniques Click here for file
