# Supplementary material for: Current approaches to gene regulatory network modelling
Source: BMC Bioinformatics. 2007 Sep 27;8(Suppl 6):S9. doi: 10.1186/1471-2105-8-S6-S9 (PMC1995542; doi:10.1186/1471-2105-8-S6-S9)
Supplement: Additional File 1 — A short primer on graph theory [file 1471-2105-8-S6-S9-S1.PDF]

A short primer on graph theory

Formally a *graph G* is a pair *(V, E)*, where *V* is the set of *nodes* (sometimes called *vertices*) and *E* is the set of *edges*. An edge $e \in E$ is a pair $e = (n_1, n_2)$, where $n_1, n_2 \in V$ are nodes. Graphs can be *directed* or *undirected*. In a directed graph edges are ordered pairs of nodes and are often called *arcs*: (n_1, n_2) means an arc from n_1 to n_2 and (n_2, n_1) means an arc from n_2 to n_1. In an undirected graph the edges do not have a particular direction (i.e., if $e_1 = (n_1, n_2) \in V$ and $e_2 = (n_2, n_1) \in V$ then $e_1=e_2$ in an undirected graph). Nodes and edges can have *labels* (i.e., character strings, name, words attached to them) and *weights* (i.e., numerical values).

To model the 'real world', nodes are used to represent entities and edges various relationships between these entities. Nodes are usually depicted as dots or circles and edges as lines connecting nodes as in Figure 1A. Arrows usually represent arcs (directed edges), where the arrowheads indicate the directionality.

In a directed graph we call nodes with outgoing edges *source nodes* and nodes with incoming edges *target nodes*; for each source node we define the *target set* as the set of all its target nodes (Figure 1B). A graph where all nodes are connected to each other by an edge is called a *complete* graph. A *clique* is a subset of the nodes in a graph such that every pair of nodes are connected by an edge (i.e., a clique is complete subgraph). A graph is a *connected graph*, if there is a *path* (a sequence of nodes such that each node in the sequence is connected to the next one by an edge or an arc) from any node to any other node in the graph. A *connected component* in a graph is a subset of nodes, such that there is a path between every pair of nodes in this subset. A graph with a small number of edges (in comparison to the complete graph) is called a *sparse graph*. We can also talk about almost complete graphs and approximate cliques. Intuitively approximate cliques in a graph representing a biomolecular network correspond to *modules*.

The *degree* of a node is defined as a number of connections (edges) adjacent to this node. For a node in a directed graph we can distinguish between the number of incoming arcs (*indegree*) and outgoing arcs (*outdegree*) (Figure 1C). In many biomolecular networks the distribution of the connections seems to follow roughly the so-called power-law: a linear relationship in a log-log plot between the degree of the nodes vs. the number of

nodes that have the particular degree [1] (Figure 3 of the main article). This implies that there are few *hubs* - nodes with a large number of connections; while most nodes have a low number of connections. This property was also observed for many other real world networks, such as the Internet (connections between computers, connections between webpages), and for social networks (such as networks of acquaintances or co-authorship of articles) [1].

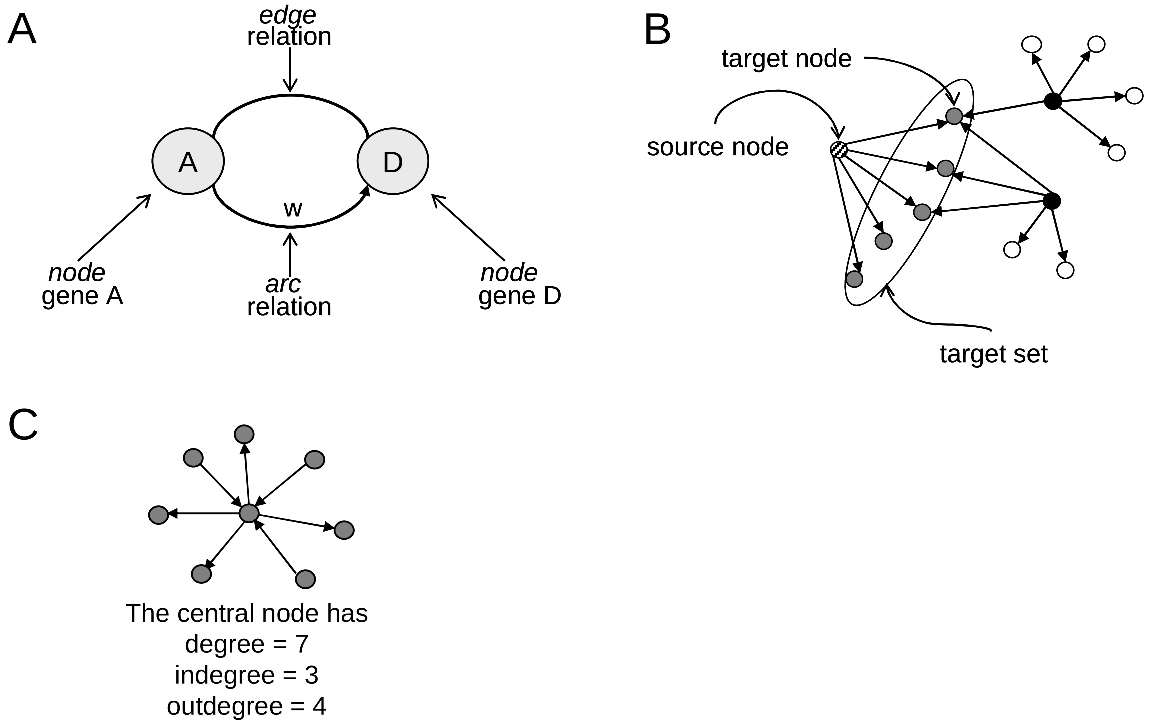

Figure 1 ***Basic elements of a graph representation.***
A Nodes are represented as circles and connections between nodes represent undirected relationships (edges) or relationships where directionality is important (arcs). **B** Source genes have outgoing arcs; target genes have incoming arcs. **C** The number of incoming and outgoing arcs is the degree of a node, the indegree is the number of incoming arcs, and the outdegree is the number of outgoing arcs. In an undirected network only the degree is defined as the number of edges of a node.

Various data structures that can be used to represent graphs [2]. For a graph $G = (V, E)$, with n nodes (i.e., $|V|=n$), an *adjacency matrix M* is an $n \times n$ binary matrix of elements m_{ij} $(i, j = 1, \ldots, n)$, such that $m_{ij} = 1$, if there is an edge from node i to j, and $m_{ij}=0$ otherwise. For a directed graph it is sufficient to represent only source nodes along one dimension and target nodes along the other dimension, i.e., $m_{ij} = 1$ if there is an arc from

source node *i* to target node *j*. For weighted graphs the adjacency matrix can be used to represent the weights of the respective edges (0 still means that there is no edge). An *adjacency list* links each source node to an array of target nodes. Graphs can also be represented by a table (*relation*) where the first column contains the source node and the second column the corresponding target node, further columns might be used to store additional information about the edge, such as its weight, etc. Each data structure has its own advantages and disadvantages. Adjacency matrices allow storing additional information about the edges, but may be memory inefficient if the network is sparse. Adjacency lists are memory efficient for sparse graphs, but make it more difficult to represent additional information (labels) related to edges.

Given a graph, various algorithms can be used to study its properties. There are efficient algorithms that allow finding *shortest path* between a pair of nodes (Dijkstra's algorithm, Bellman-Ford algorithm) or all pairs of nodes (Floyd-Warshall algorithm, Johnson's algorithm). It has been observed that most graphs with the degree distribution following a power-law have a short average path lengths between any pair of nodes [2]. Examples of other problems important for biolomolecular networks are finding cliques or approximate cliques, as these naturally correspond to 'modules' in biomolecular networks (on a topological level – see main text). Unfortunately finding cliques in a graph is an NP-hard problem, meaning that no efficient algorithm is known or likely to exist. This does not mean however that efficient heuristic algorithms working well for most real world networks do not exist. For more about graph algorithms see [1, 2, 3].

1. Albert R, Barabási A-L: **Statistical mechanics of complex networks**. *Reviews of Modern Physics* 2002, **74**(47).
2. Cormen TH, Leiserson CE, Rivest RL: **Introduction to Algorithms**. Cambridge, Mass.: MIT Press; 2001.
3. Bornholdt S, Schuster HG (eds.): **Handbook of Graphs and Networks**, 1 edn. Weinheim: Willey-VCH; 2003.
