# Supplementary material for: Current approaches to gene regulatory network modelling
Source: BMC Bioinformatics. 2007 Sep 27;8(Suppl 6):S9. doi: 10.1186/1471-2105-8-S6-S9 (PMC1995542; doi:10.1186/1471-2105-8-S6-S9)
Supplement: Additional File 2 — A very short primer on biology techniques [file 1471-2105-8-S6-S9-S2.PDF]

A very short primer on molecular biology techniques

Microarrays on single gene deletion mutants

There are different technological platforms for microarrys. Often microscope glass slides are used as a carrier for thousands of DNA spots (Figure 1). Each spot contains DNA that either corresponds to a gene (for expression studies) or a genomic region (genomic tiling arrays) (Figure 2). Hughes et al. performed an experiment using 300 different yeast deletion mutants [1]. Each yeast mutant carried one or two dysfunctional genes. Using microarrays Hughes et al. compared the gene expression activity (mRNA concentrations) of mutant versus wild type yeast, and also between wild-type and wild-type to measure systematic and random activity variation for each gene. They used the wild-type comparisons in an error-model, which allows them to calculate a gene-specific standard deviation estimate (which we denote here with γ). Although microarrays are a great tool,

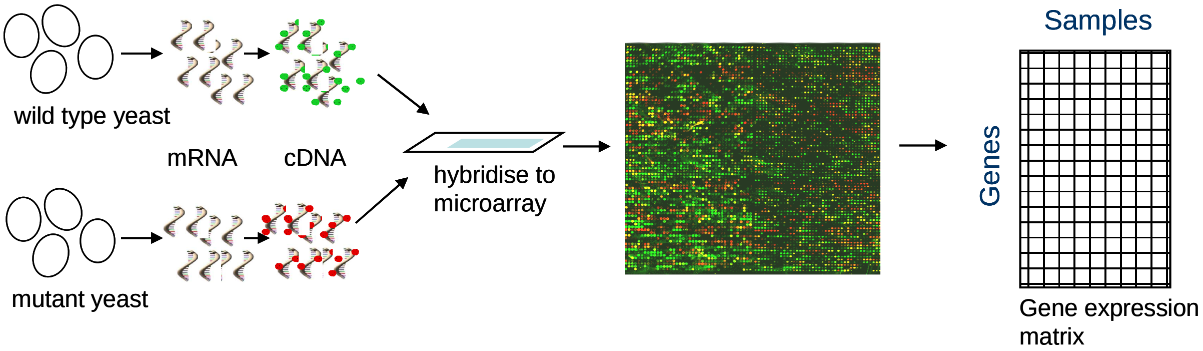

Figure 1 ***Microarray experiment.***
Two yeast cultures are grown, one with a mutant strain one with a wild type strain. Their mRNA is isolated, transcribed into cDNA and labelled with different fluorescent dyes, mixed and hybridised to a microarray. Each spot on the mircoarray corresponds to a gene, it fluorescence reflects the relative mRNA concentrations. The mircoarray is scanned and the resulting intensity values are stored in a gene expression matrix.

we have to stress that there are problems concerning specificity, accuracy, reproducibility and the biology. We can only list a few examples here, but many problems have been addressed and assessed in the literature [2, 3]. If an mRNA is present in relatively high abundance it can probably be detected reliably, however, low-copy numbers lead to problems. Cross-hybridization is likely to be common and adds to the noise in the measurements. One has to keep in mind that microarrays only measure the mRNA concentration, but mRNA half-life is sequence dependent and varies widely. Some

mRNAs are being stored for a long time until needed. Therefore, a high concentration of a mRNA does not necessarily mean that the corresponding gene is active; the concentration of a particular mRNA might not be correlated with the concentration of the corresponding protein, nor the concentration of the active form of the protein, because all posttranscriptional steps can be regulated individually. Draghici *et al*. discuss potential sources for inaccuracies in detail [3].

ChIP-on-chip technology

Transcription factor localisations can be identified experimentally using Chromatin Immuno-Precipitation (ChIP) (Figure 2B) [4]. Proteins bound to the DNA are cross-linked to the DNA permanently by treatment with formaldehyde. The DNA-protein complexes (the chromatin) are then isolated and particular DNA-protein complexes are purified (precipitated) by antibodies specific to a transcription factor (this is the “immuno” part). This procedure enriches the DNA regions bound by the transcription factor of interest. These DNA fragments are then identified for example by sequencing or with genomic tiling microarrays (ChIP-on-chip technology) [5]. Different types of genomic tiling arrays are in use; they either contain long regions of genomic DNA or contain a large number of small sequences (oligos). In either case, assigning intragenic regions to promoters of particular genes that they control is not a trivial problem, because of the large size of intragenic regions and potential long-range effects of promoters and enchancers. In the case of long genomic fragments the size of the genomic regions on the chip limit the resolution of the localisation to several hundred basepairs. Computational approaches are used to identify sequence motifs common to the enriched genomic regions. In the case of genomic tiling arrays using oligos, the analysis is problematical by the large number of oligonucleotides on the array, and difficulties in distinguishing signal from noise.

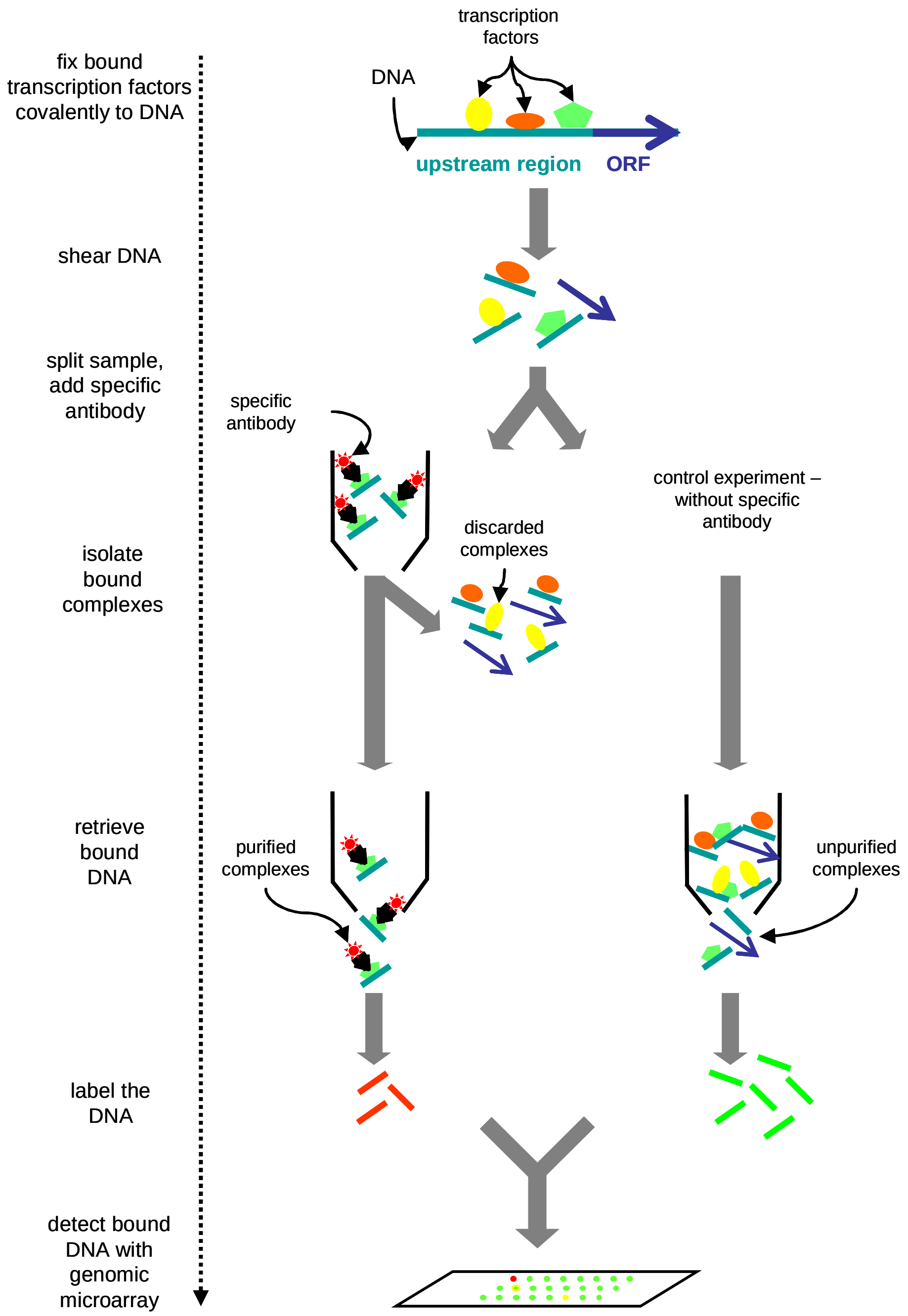

Figure 2 ***ChIP-on-chip technology.***

1. Hughes TR, Marton MJ, Jones AR, Roberts CJ, Stoughton R, Armour CD, Bennett HA, Coffey E, Dai H, He YD *et al*: **Functional discovery via a compendium of expression profiles**. *Cell* 2000, **102**(1):109-126.
2. Kothapalli R, Yoder SJ, Mane S, Loughran TP, Jr.: **Microarray results: how accurate are they?** *BMC Bioinformatics* 2002, **3**:22.
3. Draghici S, Khatri P, Eklund AC, Szallasi Z: **Reliability and reproducibility issues in DNA microarray measurements**. *Trends Genet* 2006, **22**(2):101-109.
4. Orlando V: **Mapping chromosomal proteins in vivo by formaldehyde-crosslinked-chromatin immunoprecipitation**. *Trends Biochem Sci* 2000, **25**(3):99-104.
5. Ren B, Robert F, Wyrick JJ, Aparicio O, Jennings EG, Simon I, Zeitlinger J, Schreiber J, Hannett N, Kanin E *et al*: **Genome-wide location and function of DNA binding proteins**. *Science* 2000, **290**(5500):2306-2309.
